# Co-occurrence in ant primary parasitoids: a *Camponotus rectangularis* colony as host of two eucharitid wasp genera

**DOI:** 10.7717/peerj.11949

**Published:** 2021-08-18

**Authors:** Gabriela Pérez-Lachaud, Jean-Paul Lachaud

**Affiliations:** 1 Conservación de la Biodiversidad, El Colegio de la Frontera Sur, Chetumal, Quintana Roo, México; 2 Centre de Recherches sur la Cognition Animale, Centre de Biologie Intégrative, Université de Toulouse UPS, CNRS-UMR 5169, Toulouse, France

**Keywords:** Ant-parasitoid relationships, Symbiotic associations, Local parasitism rates, Natural history, Parasitism avoidance, Polydomy

## Abstract

Different assemblages of parasitoids may attack a given host species and non-random distribution patterns in parasitoid species assemblages have been reported on various occasions, resulting in co-occurrence at the population, colony, or even individual host levels. This is the case for different closely related species of eucharitid wasps (a family of specialized ant parasitoids) sharing similar niches and co-occurring on the same host at different levels. Here we reviewed all known associations between eucharitid wasps and the ant host genus *Camponotus* Mayr, 1861 and reported new ant-parasitoid associations. In addition, we report a new case of co-occurrence in eucharitid wasps, at the host colony level, involving a new undescribed species of *Pseudochalcura* Ashmead, 1904 and an unidentified species of *Obeza* Heraty, 1985, which attack the common but very poorly known neotropical arboreal ant *Camponotus rectangularis* Emery, 1890. Most attacks were solitary, but various cocoons were parasitized by two (16%) or three (8%) parasitoids. Globally, parasitism prevalence was very low (3.7%) but showed an important variability among samples. Low parasitism prevalence along with host exposure to parasitoid attack on host plants and overlapping reproductive periods of both parasitoid species may have allowed the evolution of co-occurrence. We also provided some additional data regarding the host ant nesting habits, the colony composition and new symbiotic associations with membracids and pseudococcids. The seemingly polydomous nesting habits of *C. rectangularis* could play a part in the reduction of parasitism pressure at the population level and, combined with occasionally important local parasitism rates, could also contribute to some parts of the colonies escaping from parasites, polydomy possibly representing an effective parasitism avoidance trait.

## Introduction

Species within a community interact in different ways. Biotic interactions are extremely diverse in type, strength, or spatio-temporal scale, and can occur within or among species. Their outcome may be positive or negative and range from mutualism or facilitation to competition, parasitism or predation (Pringle, 2016). Parasitoids (arthropods whose larvae develop on or within a single host individual, ultimately killing it (Eggleton & Gaston, 1990; Godfray, 1994)) are key components of natural communities and exert strong influence on host populations (Hawkins, Cornell & Hochberg, 1997).

Because species interact over landscapes that are heterogeneous in terms of abiotic or biotic environment (Thompson, 2005), different assemblages of parasitoids may attack a particular host species throughout its distribution range. Co-occurrence (*i.e.,* the spatial overlap of the different species) may happen by chance but multiple parasitoids, including multiple obligate specialist species with similar natural history traits and specific resource requirements, may coexist on a single host (Porter & Hawkins, 2003; Pérez-Lachaud, López-Méndez & Lachaud, 2006; Pérez-Lachaud & Lachaud, 2017). Such non-random patterns of species co-occurrence are frequently observed in parasitic or microbial communities (Barberán et al., 2012; Fuhrman, Cram & Needham, 2015; Aivelo, Norberg & Tschirren, 2019; Veitch, Bowman & Schulte-Hostedde, 2020), including parasitoid species assemblages (Pérez-Lachaud, López-Méndez & Lachaud, 2006; Zhang et al., 2021). In addition to factors that influence the spatial distribution of parasitoids at the landscape and local level (Kruess, 2003; see Cronin & Reeve, 2005 for a review), the stable co-existence of multiple parasitoid species attacking the same host species is generally explained (but see Berry & Widder, 2014; Freilich et al., 2018; Blanchet, Cazelles & Gravel, 2020) by spatial or temporal niche differentiation (Zhang et al., 2021). Parasitoid coexistence usually involves differences between competitors in at least some of their natural history traits, allowing them to either exploit different sources or to exploit the same resource but using spatial or temporal differences in their attacks (Hassell, Comins & May, 1994; Comins & Hassell, 1996; Amarasekare, 2000; Slone & Allen, 2005; Zhang et al., 2021). Theoretical and empirical evidence suggests that species with similar ecological requirements tend to interact strongly, leading to negative species interactions or competitive exclusion (Godfray, 1994). However, in the case of phylogenetically related specialist parasitoids that attack the same host species and the same developmental stage, the differences between competitors may be very small or subtle and, therefore, more difficult to specify (Slone & Allen, 2005). Such co-occurrence of specialized ant parasitoids has already been reported at the population and colony levels for closely related eucharitid wasp species sharing similar niches (Pérez-Lachaud et al., 2006; Pérez-Lachaud & Lachaud, 2014), and even in the same individual ant host (multiparasitism) (Pérez-Lachaud, López-Méndez & Lachaud, 2006).

The wasp family Eucharitidae (Hymenoptera: Chalcidoidea) stands out because all species for which the host is known parasitize ants, and because females do not oviposit directly on/in the host (Heraty, 2002; Lachaud & Pérez-Lachaud, 2012). Females lay eggs in/on plants visited by the foragers of their host (Clausen, 1940b). The mobile planidium (first larval instar) gains access to the host nest either through phoresy on these foragers or on prey items that they carry (Clausen, 1923; Clausen, 1940a; Carey, Visscher & Heraty, 2012; Herreid & Heraty, 2017). Inside the nest, planidia attach to host larvae but do not kill the host immediately (koinobiosis) and complete development only on the pupae (Lachaud & Pérez-Lachaud, 2012). Newly emerged adults are not immediately aggressively treated by their hosts, although wasps can be recognized as intruders and rapidly discarded from the nest (Vander Meer, Jouvenaz & Wojcik, 1989; Pérez-Lachaud et al., 2015; Pérez-Lachaud et al., 2019b). Solitary development is the rule, but in some species several planidia (up to eleven) can be found on the same host larva (Pérez-Lachaud et al., 2010) and up to four individuals may complete their development on a single host (Wheeler, 1907; Clausen, 1923; Heraty & Barber, 1990; Pérez-Lachaud et al., 2006; Torréns, Heraty & Fidalgo, 2008; Pérez-Lachaud et al., 2010).

Obligate biotic interactions are particularly exposed to coextinction due to habitat loss and habitat fragmentation which may trigger cascades of secondary extinctions (Dunn et al., 2009; Lafferty, 2012; Brodie et al., 2014; Dallas & Cornelius, 2015). This is typically the case of ant-myrmecophile associations, especially those of neotropical arboreal ants whose colonies and nests have been identified as reservoirs of unknown myrmecophile diversity (*e.g.*, Pérez-Lachaud & Lachaud, 2014; Rocha, Lachaud & Pérez-Lachaud, 2020), although symbionts in such environments show low incidence. As part of an ongoing project on ants and their associates, here we focused on the poorly known neotropical arboreal ant *Camponotus* (*Myrmocladoecus*) *rectangularis* Emery, 1890 (Hymenoptera: Formicidae: Formicinae). Notwithstanding that *C. rectangularis* is very common in some habitats such as dry lowlands in Costa Rica (AntWeb, 2020), and is frequently mentioned in local and regional diversity studies of ants in the Neotropics, only a handful of studies have focused on this species and almost nothing is known on its associated fauna. Our study expands our scarce knowledge on ant associates and co-occurrence of specialized ant parasitoids. We also provide new records of plants serving as nesting sites and some characteristics of the composition of their colonies.

## Materials & Methods

### Ant host natural history

*Camponotus rectangularis* is an arboreal neotropical ant species consisting of six subspecies widely distributed from Sinaloa and Nuevo León in northern Mexico to Bolivia and the states of Goías and Minas Gerais in southern Brazil (Janicki et al., 2016; Guénard et al., 2017; AntWeb, 2020). Workers have a body size varying from 4.3 to nine mm (Emery, 1890; Rico-Gray & Thien, 1989) and are easily recognizable by their orange red to brown color and the distinctive rectangular shape of their propodeum.

Colonies opportunistically nest in preformed cavities of various tree species, in dead branches and stems, and even in abandoned arthropod made structures (*e.g.*, Wheeler, 1934; Durou et al., 2002; Bouwma, Howard & Jeanne, 2007; AntWeb, 2020). Quite commonly associated with epiphytes (Skwarra, 1934; Dejean, Olmsted & Snelling, 1995; Durou et al., 2002), they have been reported to establish a mutualistic association with the orchid *Myrmecophila tibicinis* (Boneman ex Lindley) Rolfe, 1838 (referred to as *Schomburgkia tibicinis*) (Rico-Gray, 1989; Rico-Gray & Thien, 1989).

Workers are active diurnally on low vegetation and tree trunks but are also commonly found in the canopy (Vergara-Torres et al., 2017). They forage for nectar produced by extra-floral nectaries and orchids (Rico-Gray, 1989; Rico-Gray, 1993) and for honeydew from aphids (as *Aphis craccivora* Koch, 1854 and *Myzocallis discolor* (Monell, 1879); Espadaler, Pérez Hidalgo & Villalobos Muller, 2012), and membracids (as *Aconophora ferruginea* Fowler, 1895; Wood, 1984). As many other *Camponotus* species, *C. rectangularis* can be considered as a true omnivore, preying on other arthropods (Young et al., 1986; Catzim, 2015) and scavenging on carrion (Cornaby, 1974). The uropodid mite *Oplitis pennsylvanica* (Berlese, 1903) has been known to be associated with this species for a long time (Skwarra, 1934), and the endosymbiotic *γ*-3 proteobacteria Candidatus *Blochmannia* Sauer et al., 2000 was documented in the midgut epithelium of the subspecies *C. r. rubroniger* Forel, 1899 more than 100 years ago (Buchner, 1918). However, no other symbiotic association has ever been recorded for this species.

### Study site, sampling, and host and parasitoids identification

Ants were collected in a 2,000 m^2^ coastal lagoon private site, located at Laguna Guerrero (18°41′20″N, 88°15′55″W), in the southern portion of Quintana Roo, Mexico, near the border with Belize (Fig. S1A). The vegetation of the site consists mainly of mangrove (*Rhizophora mangle* Linnaeus, 1753 (Rhizophoraceae) and *Laguncularia racemosa* (Linnaeus) C.F. von Gärtner, 1807 (Combretaceae)), indigenous trees (*Coccoloba uvifera* (Linnaeus) Linnaeus, 1759 (Polygonaceae), *Guazuma ulmifolia* Lamarck, 1789 (Malvaceae), *Leucaena leucocephala* (Lamarck) de Wit, 1961 and *Lysiloma latisiliquum* (Linnaeus) Bentham, 1975 (Fabaceae), *Manilkara zapota* (Linnaeus) P. Royen, 1953 (Sapotaceae), *Piscidia piscipula* (Linnaeus) Sargent, 1753 (Fabaceae)), and indigenous palm trees (*Thrinax radiata* Loddiges ex Schultes & Schultes, 1830 (Arecaceae)) intermixed with coconut palm trees (*Cocos nucifera* Linnaeus, 1753 (Arecaceae)) and ornamental plants (*e.g.*, black bamboo *Phyllostachys nigra* (Loddiges ex Lindley) Munro, 1868 (Poaceae) and *Hibiscus syriacus* Linnaeus, 1661 (Malvaceae)) (see Figs. S1B–S1D). Six complete colonies (CC) or colony fragments (CF) of *C. rectangularis* were collected between March and July 2020. However, despite an intensive search for additional *C. rectangularis* colonies in our study area (more than 80 h of surveying), we have not been able to find a single new nest since August 2020. Nesting sites were found by following foragers returning back to their nest. Three complete colonies were obtained: one (Table 1, #2CC) was established in a decaying *M. tibicinis* pseudobulb; a second (Table 1, #3CC) was collected from a *G. ulmifolia* live tree attacked by termites; finally a third (Table 1, #6CC), apparently complete colony was collected using two trap nests made of bamboo internodes (see Fig. S2 for additional information on these artificial nests) set at a height of 1.60 and 2.0 m, respectively, in a black bamboo (*P. nigra*), where ants had been previously observed foraging, and let in place during four weeks. In addition, three colony fragments were collected: one (Table 1, #1CF) in a hollow dead branch of a *G. ulmifolia* live tree, at 1.2 m height; another (Table 1, #4CF) in a hollow dead branch of *P. piscipula*, hanging at a height of 1.7 m in the black bamboo environment; a third (Table 1, #5CF) at the cut end of a native palm leaf petiole (*T. radiata*).

**Table 1:** Composition of the samples of *Camponotus rectangularis* collected at Laguna Guerrero, Quintana Roo, Mexico, and results of the cocoons dissection.

								Parasitized		
	Q	G	Males	Workers	Pupae	Larvae	Eggs		Number of cocoons		Composition			Parasitism rate (%)	
#1CF March 29				18	17	112		5 (1 triple, 1 double, 3 simples)			triple: 2 ♂**+** 1 ♀*Pseudochalcura* sp. /puW double: 1 ♂**+** 1 ♀*Pseudochalcura* sp. /puW simple: 1 ♀, 1 ♂*Pseudochalcura* sp. /puW, 1L_2_ /puW			29.4	
#2CC April 30	1			246	137	367	+++		10 simples			simple: 5 ♀, 1 ♂*Pseudochalcura* sp. /puW, 1 ♀*Obeza* sp. /puW, 1L_2_ /puW, 2 planidia /ppu			7.3	
#3CC May 14	1	7	277	535	468	189	+++		2 simples			simple: 1 ♀*Pseudochalcura* sp. /puW; 1 planidium /ppu			0.4	
#4CF June 21		16	2	35	15	11		0			-			0	
#5CF July 11		10		13		1		NA			-			0	
#6CC July 29	1 ^∗^	53	6	105	33	29		8 (3 doubles, 1 triple, 4 simples)			triple: 3L_3_ /puW double: 2L_3_ /pu ♂, 2L_3_ /pu ♂, 1 pu ♂*Pseudochalcura* sp. **+** 1L_3_ /pu ♂ simple: 2L_3_ /pu ♂, 1L_2_ /pu ♂**,** 1 fully fed L_1_ /ppu			24.2	

**Notes.**

* The dealate female was dissected and found devoid of mature eggs.

# sample identification CC complete colony CF colony fragment Q dealate females G unmated alate females (gynes) ppu unidentifiable prepupa pu pupa /ppu on ant prepupa (unidentified caste) /puW on worker ant pupa /pu ♂ on male ant pupa

The nests were broken open and all ants and organisms present were collected. For each colony or colony fragment, we recorded the number of queens, gynes (alate females), males, workers, cocoons, and larvae, while the quantity of eggs was globally assessed; any other organism present was also noted. Adults were placed in 96% alcohol and cocoons and larvae were kept with several workers in glass vials stuffed with cotton (at 28 ± 2°C and 75 ± 5% RH) for about one week while awaiting adult parasitoid emergence, if any. After this period, all the material was preserved in 96% alcohol and examined under a Nikon SMZ-745T stereomicroscope. The larvae were thoroughly revised for the presence of any planidium or evidence of parasitoid attack (scars evidencing a previous unsuccessful attack or signs of endoparasite presence, see Pérez-Lachaud & Lachaud, 2014; Pérez-Lachaud et al., 2017). The cocoons were carefully dissected and their contents—both the host ant remains and the parasitoid(s) when present—were identified whenever possible. This allowed us to ascertain to which caste (female, male, or worker) the host belonged, even if only the exuviae remained. The number and developmental stage of the parasitoids (planidia, fully fed L_1_, L_2_, L_3_, pupae, and pharate adults) were recorded. Adult ants were also closely examined for the presence of potential ecto- or endoparasites (*e.g.*, phorid flies, strepsipterans, mites, nematodes, or planidia) attached to their body.

The ants were identified using resources on AntWeb (2020) and compared with specimens from the Formicidae collection of El Colegio de la Frontera Sur (ECO-CH-F: DF-CC-289-15) which provides a large sample of the ant species of Quintana Roo (see Lachaud & Pérez-Lachaud, 2013). Eucharitids were identified to genus level with appropriate taxonomic keys (Heraty, 1985; Heraty, 1986; Heraty, 1997; Heraty, Heraty & Torréns, 2009; Torréns, 2016) and confirmed by John Heraty and Scott Heacox based on morphology and molecular data (sequencing of the 28S-D2 region) obtained as part of an independent study. Other organisms associated with *C. rectangularis* were identified to order or family level. Voucher specimens of ants and parasitoids were deposited in the Formicidae and Arthropoda collections of El Colegio de la Frontera Sur at Chetumal, Quintana Roo, Mexico (ECO-CH-F and ECO-CH-AR, respectively) and in the collection of the Entomological Research Museum of the University of California, Riverside (vouchers: UCRC_ENT00468545 (adult female); UCRC_ENT00468551 (pupa)). Field sampling comply with the current laws of Mexico and was carried out under permit number FAUT-0277 issued by the Secretaría de Medio Ambiente y Recursos Naturales, Dirección General de Vida Silvestre (Secretary of Environment and Natural Resources of Mexico).

## Results

### Colony composition and within-nest associated organisms

**Figure 1:**
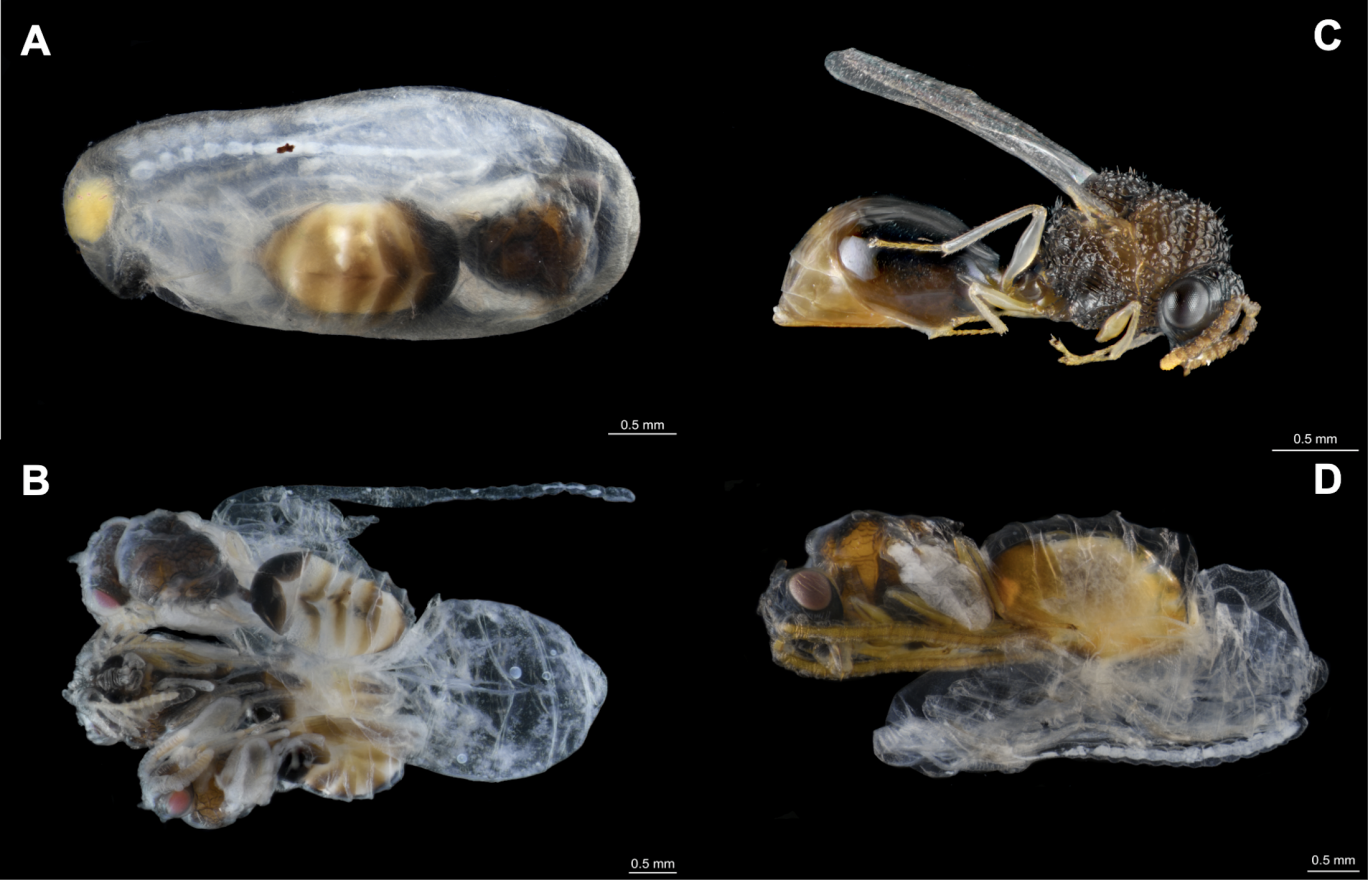
Eucharitid parasitoids of *Camponotus rectangularis*. (A) *Pseudochalcura* sp. female pupa visible by transparency through the silk cocoon. (B) Gregarious development of *Pseudochalcura* sp. (two males, one female); the cocoon has been removed. (C) *Pseudochalcura* sp. female adult. (D) *Obeza* sp. female pupa almost completely pigmented; the cocoon has been removed. Photos credit: Humberto Bahena-Basave.

**Figure 2:**
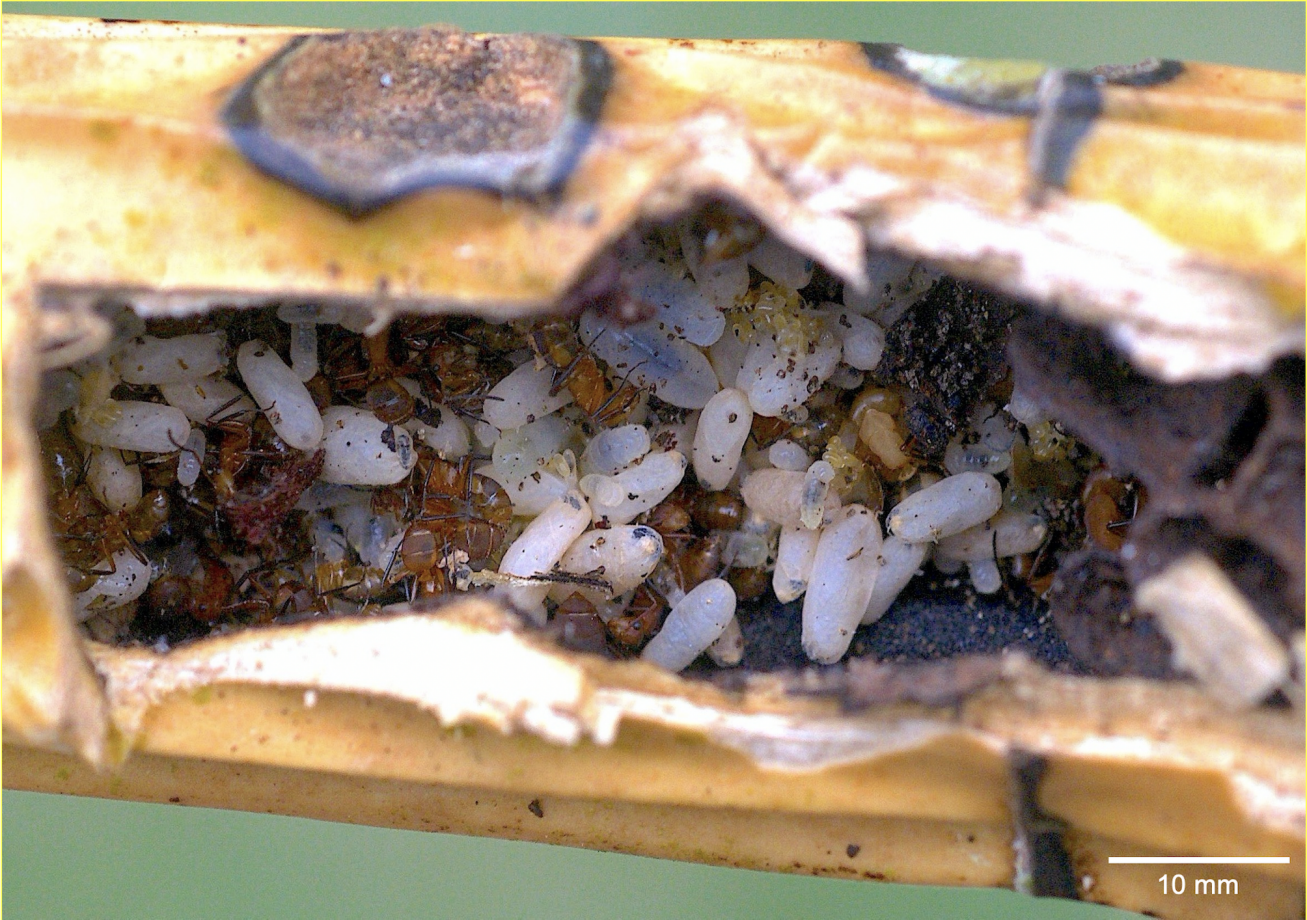
A *Camponotus rectangularis* nest established in a decaying pseudobulb of the orchid *Myrmecophila tibicinis*. Photo credit: Jean-Paul Lachaud.

The first colony fragment nested in a hollow branch along with a *Crematogaster crinosa* Mayr, 1862 colony. Out of 17 cocoons, five were parasitized by eucharitid wasps (Table 1, #1CF): three by a single parasitoid, one by two parasitoids and one by three parasitoids (Figs. 1A, 1B, 1C). With the exception of one L_2_ which died and was preserved, seven parasitoids attained adulthood (three females, four males) and were identified as belonging to the genus *Pseudochalcura* Ashmead, 1904 (Eucharitinae). Molecular data and morphology confirmed this species as new to science (J. Heraty, 2021, personal correspondence). A complete colony was found in the pseudobulb of *M. tibicinis* (Fig. 2), separated from a large unidentified juvenile spider (possibly Miturgidae or Liocranidae) by a carton structure made by the ants at the base of the pseudobulb and numerous *Pseudomyrmex* spp. remains were found in the refuse pile of the colony. Out of 137 cocoons, 10 were parasitized, all of them by a single parasitoid (Table 1, #2CC): six by adults of *Pseudochalcura* sp. (five females, one male), one by a female (in the pupa stage) of an unidentified species of *Obeza* Heraty, 1985 (Fig. 1D, Fig. S3D), and three were immature stages (two planidia and one L_2_) which could not be ascribed to any of the two eucharitid species. A second complete colony was larger and contained 468 cocoons but, perhaps due to the poor preservation conditions of the material (51 cocoons were crushed and attacked by fungi), only two were found parasitized by a single parasitoid (Table 1, #3CC): a female *Pseudochalcura* sp. (Fig. S4B) and a fully fed L_1_ (Fig. S3A). A second colony fragment contained 15 cocoons, but none was parasitized, and a third colony fragment had no cocoon (Table 1, #4CF, #5CF, respectively). Finally, a third apparently complete colony contained 33 cocoons, out of which eight were parasitized (Table 1, #6CC): four by single parasitoids (all immature stages, Fig. S3C), three by two (which yielded one male pupa of *Pseudochalcura* sp. and five L_3_, Fig. S3B), one by three (three L_3_, Fig. S4A). A thorough examination of all the adult ants (three dealate females, 86 gynes, 285 males, 952 workers) and larvae (709) from the six colonies did not reveal any evidence of parasitism by eucharitids or any other endo- or ectoparasite or parasitoid, except for the presence of phoretic mites (Mesostigmata, Laelapidae) on various gynes of one of the sub-units of sample #6CC.

Dissection of the two wingless females found in samples #2CC and #3CC showed that their abdomen was filled with well-developed eggs. Sample #6CC contained a large number of gynes (Table 1) and one dealate female but, after dissection, this dealate female resulted devoid of mature oocytes suggesting that she was merely an unmated female who had recently lost her wings. Therefore, the colony distributed in the two artificial nests appeared not to be a complete colony but a fragment that had migrated from a larger colony probably nesting higher in the black bamboo. The adult population size of the two queenright colonies of *C. rectangularis* collected varied from 247 to 820, with only one queen but numerous eggs, larvae and pupae (Table 1). The number of adults and brood was much more reduced and variable in colony fragments, but occasionally numerous larvae and reproductive adults were found (Table 1).

### Parasitism rate

As first highlighted by Heraty & Barber (1990) for *P. gibbosa* (Provancher, 1881), the planidia of the new *Pseudochalcura* species observed attacking *C. rectangularis* departed from the general form of eucharitid planidia (see Clausen, 1923; Clausen, 1928; Clausen, 1940a; Heraty, 2002) with a reduced number of tergites (five instead of twelve). In addition, in the case of our *Pseudochalcura* species, the tergites were only slightly sclerotized (Fig. S3A) and anchoring into the host did not produce an apparent scar. This made it much more difficult to detect initial stages of parasitism and some might have gone unnoticed; consequently, the parasitism rate reported here below is possibly an underestimation.

In total 33 eucharitid wasps were obtained from 25 parasitized cocoons. Most attacks were solitary (76%), but various cocoons were parasitized by two (16%) or three (8%) parasitoids. Globally, the prevalence of parasitism for the studied population was only 3.7%; however, there was an important variability in the parasitism rate among the samples (range: 0.0–29.4%). The targets of the 21 attacks observed for which the caste of the host could be identified involved both worker (15 cases) and male (six cases) host pupae while female pupae were not parasitized. This appeared to be independent of the number of available host cocoons for each caste as suggested by the absence of parasitism of female cocoons in the last collected sample where gynes were nevertheless numerous (Table 1, #6CC). However much more sampling effort would be needed to assess whether this caste preference is significant and could really apply to the whole population.

### Associated trophobionts outside the nests

**Figure 3:**
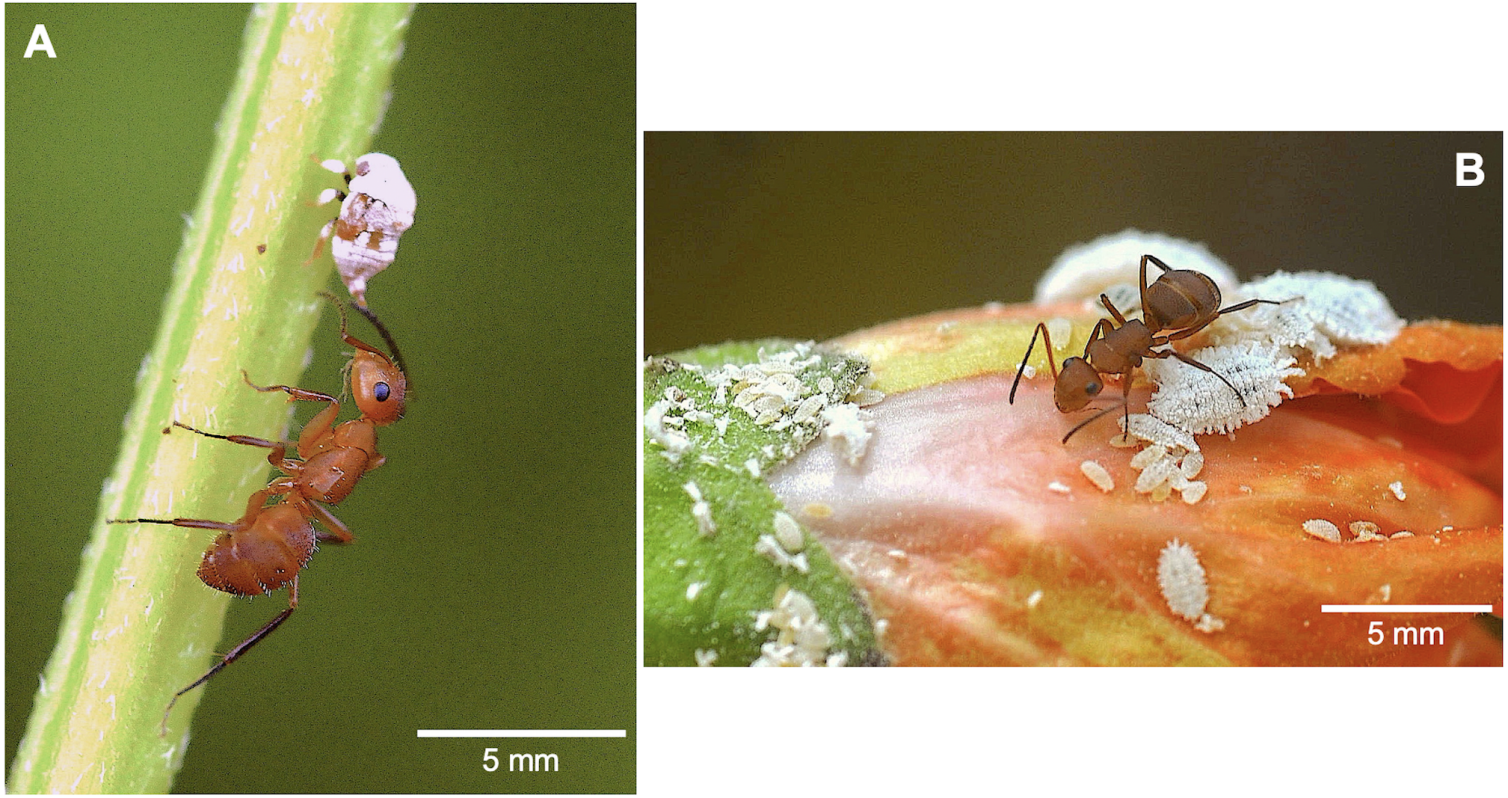
*Camponotus rectangularis* workers collecting honeydew. (A) From an unidentified membracid nymph. (B) From *Planococcus citri* mealybugs on *Hibiscus syriacus*. Photos credit: Jean-Paul Lachaud.

In addition to the within-nest associations reported above, two other associations were recorded outside the nests, involving trophobionts. *Camponotus rectangularis* workers were observed climbing to *L. leucocephala* and *P. piscipula* trees to forage in the canopy, but they also collected honeydew produced by Hemiptera on ornamental plants (for example, from unidentified membracid nymphs (Fig. 3A) and pseudococcids (*Planococcus citri* Risso, 1813, Fig. 3B).

## Discussion

### Co-occurrence in specialized ant parasitoids

Our results contribute to the knowledge on ant-associated organisms and draw attention to co-occurrence in specialized ant parasitoids. Two new eucharitid-host associations and a new case of co-occurrence at the host colony level are reported here, involving an undescribed species of *Pseudochalcura* new to science (J. Heraty, 2021, personal correspondence) and an unidentified species of *Obeza*, which attack the common but very poorly known neotropical arboreal ant *C. rectangularis*. Eucharitids are koinobionts, initially attacking ant larvae but completing their development on the pupae. They develop mainly as solitary ectoparasitoids but up to four individuals may attain adulthood from the same individual host, depending on the size and caste of the targeted ants attacked (Lachaud & Pérez-Lachaud, 2012). A single individual, a female pupa, was obtained for *Obeza*; however, out of a total of 15 individuals of *Pseudochalcura* whose sex could be identified, nine were females and six were males, accounting for a relatively balanced sex-ratio in this species. Most eucharitid attacks on *C. rectangularis* (76%) were solitarily; however, for *Pseudochalcura* sp. six cases of gregarious development occurred (four double, two triple) and different developmental stages were found in one of them suggesting differential development rate or differential attack time. This is the first instance of parasitism of *C. rectangularis* by two species from two different genera of eucharitid wasps which, furthermore, can attack the same host colony, evidencing a new case of co-occurrence in ant primary parasitoids.

Co-occurrence is a phenomenon widely present in parasites and, in some instances, the interaction of several parasites in a single host may facilitate the attack as recorded for co-occurring infections (Dallas, Laine & Ovaskainen, 2019; Veitch, Bowman & Schulte-Hostedde, 2020). However, co-occurrence in parasitoids is rare (Hawkins, Cornell & Hochberg, 1997), especially in the case of parasitoids sharing similar niches or the same host, such as eucharitids. While specific species of ants are commonly attacked by different parasitoid species along their distributional range, including humpbacked flies (Phoridae), wasps of the Eulophidae, Diapriidae and Eucharitidae families (Lachaud & Pérez-Lachaud, 2012; Folgarait, 2013; Elizalde et al., 2018), and mites (Pérez-Lachaud et al., 2019a), the co-occurrence of two or more ant parasitoid species in the same host colony is rare, in particular for eucharitid parasitoids. Our study on *Camponotus rectangularis* is only the second case of such co-occurrence of two eucharitid species at the colony level, a previous case having been reported in *Ectatomma tuberculatum* (Olivier, 1792), involving *Isomerala coronata* (Westwood, 1874) and *Dilocantha lachaudii* Heraty, 1998 (Pérez-Lachaud, López-Méndez & Lachaud, 2006; Pérez-Lachaud et al., 2010). According to Hughes, Pierce & Boomsma (2008), specialized parasites of long-lived insect societies tend to be less virulent than those associated with non-social hosts. As eucharitids get access to the host nest through phoresis on foraging ant workers, it is possible that *C. rectangularis* foragers were exposed to both *Pseudochalcura* and *Obeza* planidia, maybe on the same host plants (not identified in this study) and during the same time window. Despite the relatively low attack rate at the population level of specialized ant parasitoids, both the host exposure to planidia attack on host plants where ants forage and the overlapping of reproductive periods of the parasitoids may have allowed the evolution of co-occurrence in eucharitid wasps.

Females of *Obeza* have been reported to oviposit into small berries such as the fruits of *Vaccinium simulatum* Small, 1903 (Ericaceae) used by *O. floridana* (Ashmead, 1888) in Florida (Heraty & Barber, 1990) while most of the records obtained for *Pseudochalcura* indicated that females of this genus oviposit preferentially into developing flower buds (*Gossipium thurberi* Todaro, 1877 (Malvaceae) in Arizona (Pierce & Morrill, 1914) and *Rhododendron groenlandicum* (Oeder) Kron & Judd, 1990 (Ericaceae) in northern Ontario (Heraty & Barber, 1990) for *P. gibbosa*; *Eryngium* sp. (Umbelliferae) in Uruguay (Heraty, 1986) for *P. nigrocyanea* Ashmead, 1904). Females of *P. gibbosa* can also oviposit on leaf buds of *Arbutus menziesii* Pursh, 1813 (Ericaceae) in northern Ontario (Heraty & Barber, 1990) and have been reported on various plants (*Pinus jeffreyi* Balfour, 1853, *Pinus ponderosa* C. Lawson, 1836, and *Larix laricina* (Du Roi) K. Koch, 1873 (Pinaceae) in California and Wisconsin, *Chrysothamnus* sp. (Asteraceae) in Nevada, and *Rhus glabra* Linnaeus, 1753 (Anacardiaceae) in New Mexico (Heraty, 1986). Finally, a female of *P. condylus* Heraty, 1986 has been collected on *Varronia curassavica* Jacquin, 1760 (Boraginaceae) in Trinidad (Heraty, 1986). Undoubtedly, eucharitid females in our study site oviposit in some of the resources that the host foragers visit, and plants of the family Malvaceae could be good candidates. Unfortunately, *C. rectangularis* workers foraged mainly in the tree canopy, which at our study site was about 10-15 m in height, and we were unable to witness any oviposition behavior.

**Table 2:** Known associations of eucharitid wasps with species of the ant genus *Camponotus*.

**Eucharitidae**	**Referred to as**	**Host(s)**	**Referred to as**	**Locality**	**References**
*Ancylotropus manipurensis* (Clausen)^*^		*Camponotus* sp.		India	Narendran & Sheela (1995)
	*Stilbula*	*Camponotus* sp.		India	Clausen (1941) (p. 58)
	*Stilbula manipurensis*	*Camponotus* sp.		India	Heraty & Barber (1990) (p. 249)
*Hydrorhoa* sp. *striaticeps* complex		*Camponotus maculatus* (Fabricius)	*C. maculatus* Mayr	South Africa	Heraty (2002) (p. 161)
*Lophyrocera variabilis* Torréns, Heraty & Fidalgo		*Camponotus* sp.		Argentina	Torréns, Heraty & Fidalgo (2008) and Torréns (2013)
*Mateucharis rugulosa* Heraty		*Camponotus* sp.		Tanzania	Heraty (2002) (p. 199)
*Obeza floridana* (Ashmead)		*Camponotus floridanus* (Buckley)	*C. abdominalis floridanus* (Buckley)	USA: Florida	Davis Jr & Jouvenaz (1990)
*Obeza* sp.		*Camponotus* sp. ca. *textor* Forel		Mexico: Chiapas	Pérez-Lachaud & Lachaud (2014)
*Obeza* sp.1		*Camponotus atriceps* (F. Smith)		Mexico: Chiapas	De la Mora et al. (2015)
*Obeza* sp.2		*Camponotus rectangularis* Emery		Mexico: Quintana Roo	This work
*Orasema* sp.^**^		*Camponotus ocreatus* (Emery)		USA: Arizona	Herreid & Heraty (2017)
		*Camponotus* sp.		USA: Arizona	Herreid & Heraty (2017)
*Pseudochalcura americana* (Howard)		*Camponotus* sp. ca. *textor* Forel		Mexico: Chiapas	Pérez-Lachaud & Lachaud (2014)
*Pseudochalcura gibbosa* (Provancher)		*Camponotus herculeanus* (L.)		Canada: northern Ontario	Heraty & Barber (1990)
		*Camponotus laevigatus* (F. Smith)		USA: California	Heraty (1986)
		*Camponotus novaeboracensis* (Fitch)	*C. ligniperdus* var. *novaeboracensis* (Fitch)	USA: Michigan	Wheeler (1907)
		*Camponotus* sp. ?*vicinus* Mayr		USA: California	Heraty (1986)
*Pseudochalcura nigrocyanea* Ashmead		*Camponotus* sp.		Brazil	Heraty, Heraty & Torréns (2009)
*Pseudochalcura sculpturata* Heraty *Pseudochalcura* sp.		*Camponotus planatus* Roger *Camponotus rectangularis* Emery		USA: Florida Mexico: Quintana Roo	Heraty (2002) (p. 222) This work
*Rhipipalloidea mandagensis* Maeyama, Machida & Terayama		*Camponotus* (*Tanaemyrmex*) sp.		Papua New Guinea	Maeyama, Machida & Terayama (1999) (p. 306)
*Stilbula cyniformis cyniformis* (Rossi)	*Stilbula cynipiformis* (Rossi)	*Camponotus aethiops* (Latreille)		France	Parker (1932) and Parker (1937)
	*Stilbula cynipiformis* (Rossi)	*Camponotus aethiops* (Latreille)	*C. marginatus* Latr.	Austria	Fahringer & Tölg (1912)
	*Stilbula cynipiformis* (Rossi)	*Camponotus* sp.		France	Parker & Thompson (1925)
	*Stilbula cynipiformis* (Rossi)	*Camponotus sanctus* Forel	*C. maculatus* F. r. *sanctus* Forel	Austria or Turkia	Fahringer (1922)
*Stilbula cyniformis tenuicornis* (Ashmead)	*Schizaspidia tenuicornis* Ashm.	*Camponotus japonicus* Mayr	*C. herculeanus* sub-sp. *japonicus* Mayr	Japan	Clausen (1923) and Clausen (1941)
	*Schizaspidia tenuicornis* Ashm.	*Camponotus obscuripes* Mayr	*C. herculeanus* sub-sp. *ligniperdus* var. *obscuripes* Mayr	Korea	Clausen (1923) and Clausen (1941)
*Stilbula vitripennis* Masi		*Camponotus aegyptiacus* Emery		Egypt	Gadallah & Shairra (2019)
*Stilbuloida doddi* (Bingham)	*Schizaspidia doddi* Bingham	*Camponotus* sp.		Australia: Queensland	Dodd (1906) (p. 123)
*Zulucharis campbelli* Heraty		*Camponotus* sp.		South Africa	Heraty (2002) (p. 283)

**Notes.**

* Association mistakenly reported by Clausen (1941), p. 58 (as *Stilbula*), Heraty & Barber, 1990, p. 249 (as *Stilbula manipurensis*), and Narendran & Sheela (1995) (as *Ancylotropus manipurensis*) in base of the observations made by Clausen (1928) (see Heraty, 2002).

** Presence only on the mouthparts of foraging workers. Probably an accidental association as a result of the nectarivorous habits of various *Camponotus* species which could function as intermediate hosts (see Herreid & Heraty, 2017).

### *Camponotus* species as hosts of eucharitids

Our records of *Pseudochalcura* and *Obeza* provide a new *Camponotus* host for eucharitid primary parasitoids of ants in the New World. Although some studies have questioned the degree of host specificity in eucharitids and the factors that determine their association with their hosts (Pérez-Lachaud et al., 2006; Pérez-Lachaud, López-Méndez & Lachaud, 2006; Lachaud, Cerdan & Pérez-Lachaud, 2012), most eucharitid species have long been considered host-specific parasitoids, at least at the host genus level (Heraty, 1994; Heraty, 2002; Lachaud & Pérez-Lachaud, 2012). Our data tend to confirm such a specificity, at least for the eucharitid species associated with the species-rich genus *Camponotus* (Table 2). Up to now, nine genera of Eucharitidae, *Obeza*, *Hydrorhoa* Kieffer, 1905, *Stilbula* Spinola, 1811, *Stilbuloida* Bouček, 1988, *Lophyrocera* Cameron, 1884, *Mateucharis* Bouček & Watsham, 1982, *Rhipipalloidea* Girault, 1934, *Zulucharis* Heraty, 2002, and *Pseudochalcura*, have been reliably associated with *Camponotus* hosts worldwide (Table 2). All of the 27 species of ant hosts reported for species in these 9 eucharitid genera where the host is known (see Table 2, and also Lachaud & Pérez-Lachaud, 2012), belong to the genus *Camponotus*, except for three species of *Stilbula* and *Rhipipalloidea* associated with *Polyrhachis* Smith, 1857 and one of *Stilbuloida* associated with *Calomyrmex* Emery, 1895, two formicine genera belonging to the same tribe Camponotini (Ward, Blaimer & Fisher, 2016). The genus *Pseudochalcura* currently consists of 15 species that are distributed in the New World from Chile and Argentina to the Nearctic region as far north as Yukon and Alaska (Heraty, 1986; Heraty, 2002; Heraty, Heraty & Torréns, 2009; Torréns, 2016). It belongs to the *Stilbula* clade, a distinct group within the Eucharitini, with all known host records belonging to the ant genus *Camponotus* (Heraty, 2002; Heraty, Heraty & Torréns, 2009; Pérez-Lachaud & Lachaud, 2014). However, hosts have been identified at species level for only four *Pseudochalcura* species (Table 2) and, at least in the case of *P. gibbosa*, a close species-specific relationship with the host does not appear to exist. Similarly, all known hosts for the genus *Obeza* also belong to *Camponotus* (Davis Jr & Jouvenaz, 1990; Pérez-Lachaud & Lachaud, 2014; De la Mora et al., 2015), but until now only the host of *O. floridana* has been identified at the species level (Table 2). According to Quevillon & Hughes (2018), *Camponotus* (with 192 records) leads the list of the ten ant genera with the highest number of parasites (including parasitoids), although fewer than 4% of the total estimated ant species (580/16357 valid species and subspecies) have any parasitic associate recorded. Known parasitic organisms associated with *Camponotus* ants include virus, fungi (Laboulbeniaceae and Cordycipitaceae), Trematoda, Nematoda, and insect parasitoids of three orders: Diptera (Phoridae), Strepsiptera (Myrmecolacidae) and Hymenoptera (Braconidae, Eulophidae, Eucharitidae, Eurytomidae). Furthermore, *Camponotus* is the numerically leading ant genus with regards to the number of associated parasitic lycaenid butterfly species (cuckoo like social parasite and brood predators; reviewed in Fiedler, 2012). They also host a number of other well integrated myrmecophiles such as *Microdon* Meigen, 1803 syrphid flies, some diapriid wasps (Loiácono, 2000), and the myrmecophilous cricket *Myrmecophilus albicinctus* Chopard, 1924 (Chopard, 1924; Komatsu & Maruyama, 2016).

### Polydomy and parasitism pressure

Mature colonies of *C. rectangularis* appear to be monogynous (when present, only one queen has been found and its ovaries were fully developed) and frequently occupy several cavities. The rapid relocation, in less than four weeks, of large groups of adults and brood into artificial nests as occurred in one of our samples, suggests a remarkable capacity for rapid resettlement. Such a capacity would allow a major advantage for this species as cavity size and nest site availability are the most important limiting factors for arboreal cavity-dwelling ants (Fonseca, 1999; Philpott & Foster, 2005; Sagata et al., 2010; Burns et al., 2019; Novais et al., 2020) which commonly need to distribute their population into several cavities. This polydomous nesting strategy is supposed to enhance the foraging capacity of the colonies (Davidson, 1997; Cerdá, Dahbi & Retana, 2002; Debout et al., 2007; Stroeymeyt, Joye & Keller, 2017), an effect that would be reinforced by the generalist diet habits of *C. rectangularis* and the random distribution of its alimentary resources. Furthermore, polydomy in a monogynous species could promote reproductive success by evading queen control, reducing the attacks of the queen over sexual brood or triggering male laying behavior by workers (Cerdá, Dahbi & Retana, 2002; Denis et al., 2006; Giehr et al., 2020). It has also been suggested that polydomy could reduce the effects of the interference competition by other colonies (intra- or interspecific), as well as the pressure of predation or parasitism (Cerdá & Retana, 1998; Robinson, 2014; Burns et al., 2019). Parasitism pressure in *C. rectangularis* is almost unknown but our results show that attacks by eucharitid wasps could be locally important with parasitism rates reaching more than 24% in two of the six samples studied. In general, some nest-site characteristics of arboreal species, such as height or concealment, may affect the nest conspicuousness and accessibility, and differently impact its predation or parasitism according to the predator or parasite species involved (*e.g.*, Seeley, Seeley & Akratanakul, 1982; Martin, 1988; Colombelli-Négrel & Kleindorfer, 2009). However, unlike most other parasitoids, eucharitid females do not lay eggs directly in or on the host but on plants visited by host foragers, which ultimately convey the parasitoid into the host nest. Therefore, nest height and nest concealment probably do not play a significant role in reducing parasitism rate. The fact that the adults of a colony fragment in one of the two bamboo traps (sample #6CC) were found infested by unidentified Lalelapidae phoretic mites while those in the other fragment were not, seems to suggest that in *C. rectangularis*, polydomy may instead contribute to some parts of the colony escaping from parasites. Such a hypothesis should be further investigated in future studies using artificial traps of the same type as those described for our sample #6CC that are likely to promote the splitting of *C. rectangularis* colonies in several units.

## Conclusions

The arboreal ant *C. rectangularis* shows generalist diet habits and appears to be monogynous and polydomic, exhibiting a remarkable capacity for rapid resettlement in several dispersed, pre-existing cavities. In southeastern Mexico, it is attacked by two eucharitid species belonging to two different genera which can co-occur in the same host colony. Our record of *Pseudochalcura* sp. and *Obeza* sp. as guests of *C. rectangularis* constitutes both the first report of eucharitid wasps attacking this host and a new *Camponotus* host for eucharitids. Along with the presence of a species of *Pseudochalcura* new to science (J. Heraty, 2021, personal correspondence), our findings seem to confirm previous assumptions that arboreal ant colonies are reservoirs of unknown myrmecophile diversity (Pérez-Lachaud & Lachaud, 2014; Rocha, Lachaud & Pérez-Lachaud, 2020). However, as it is typical in ant societies, the prevalence of parasitism was very low at the overall population level (although it could be locally significant and have a harmful effect on specific colonies), what might have allowed for the evolution of co-occurrence in such specialized parasitoids. The seemingly polydomic nesting habits of *C. rectangularis*, along with high variability of parasitism rate among nesting units, could account for some parts of the colonies escaping from parasites. Co-occurrence in ant parasitoids is rare and this is only the second reported case of co-occurrence of two eucharitid species at the host colony level.

##  Supplemental Information

10.7717/peerj.11949/supp-1 Supplemental Information 1 Study site (Laguna Guerrero) (A) Location of the study site (Laguna Guerrero) in the southern part of the Yucatan Peninsula, Mexico. (B–D) General view of the habitat. (B) Mangrove (*Rhizophora mangle*) with epiphytes, namely *Myrmecophila tibicinis* (bottom, near center). (C) and (D) Examples of indigenous trees (*Lysolima latisiliquum*, *Manilkara zapota*, *Guazuma ulmifolia*), indigenous palm trees (*Thrinax radiata*), intermixed with coconut palm trees (*Cocos nucifera*) and ornamental plants (black bamboo, *Phyllostachys nigra*). Map credit: Holger Weissenberger. Photos credit: Jean-Paul Lachaud.

10.7717/peerj.11949/supp-2 Supplemental Information 2 Trap nests used to sample *Camponotus rectangularis* (A) Position of the two nesting units corresponding to sample #6CC. (B) Close-up of the lowest unit of sample #6CC. (C) Details of the nest entrance and the attachment of an artificial nest at another location. We used hollow bamboo internodes drilled at one end and closed with a cork pierced with a hole, which served as the entrance (yellow arrow in C). These artificial nests were then attached with wires (red arrow in C), at a heigh of at least 1.2 m, to a plant support where foragers had been previously observed and left without intervention for four weeks. Prior to the collection of sample #6CC, numerous interactions were observed between the two artificial nests, with several workers going back and forth between the two nesting units. Photos credit: Jean-Paul Lachaud.

10.7717/peerj.11949/supp-3 Supplemental Information 3 Immature stages of eucharitid parasitoids attacking *Camponotus rectangularis* (A) Unidentified fully fed planidium upon a *C*. *rectangularis* prepupa. Arrow points at the swollen planidium, barely visible. (B) Unidentified L_3_ (arrow) upon *C*. *rectangularis* pupa. (C) Unidentified late L_3_-prepupa (arrow) upon the host remains. (D) Pupa of the female *Obeza* sp. The host cocoon has been removed in A and C. Photos credit: Gabriela Pérez-Lachaud.

10.7717/peerj.11949/supp-4 Supplemental Information 4 The parasitoid *Pseudochalcura* sp. on its host *Camponotus rectangularis* (A) Three late L_3_-prepupae developing upon a single host. (B) Female, dorsal view. The host cocoon has been removed. Photos credit: Humberto Bahena-Basave.

## Additional Information and Declarations

### Field Study Permissions

The following information was supplied relating to field study approvals (*i.e.*, approving body and any reference numbers): Collecting and field work comply with the current laws of Mexico. The Secretaria de Medio Ambiente y Recursos Naturales (SEMARNAT, Secretary of Environment and Natural Resources of Mexico), approved field sampling under the collection permit FAUT-0277 granted to GP-L.

### Data Availability

The following information was supplied regarding data availability: The complete data on parasitism by the two parasitoid species studied are available in Table 1.
