# Supplementary figures and images for: Co-occurrence in ant primary parasitoids: a Camponotus rectangularis colony as host of two eucharitid wasp genera
Source: PeerJ. 2021 Aug 18;9:e11949. doi: 10.7717/peerj.11949 (PMC8380026; doi:10.7717/peerj.11949)

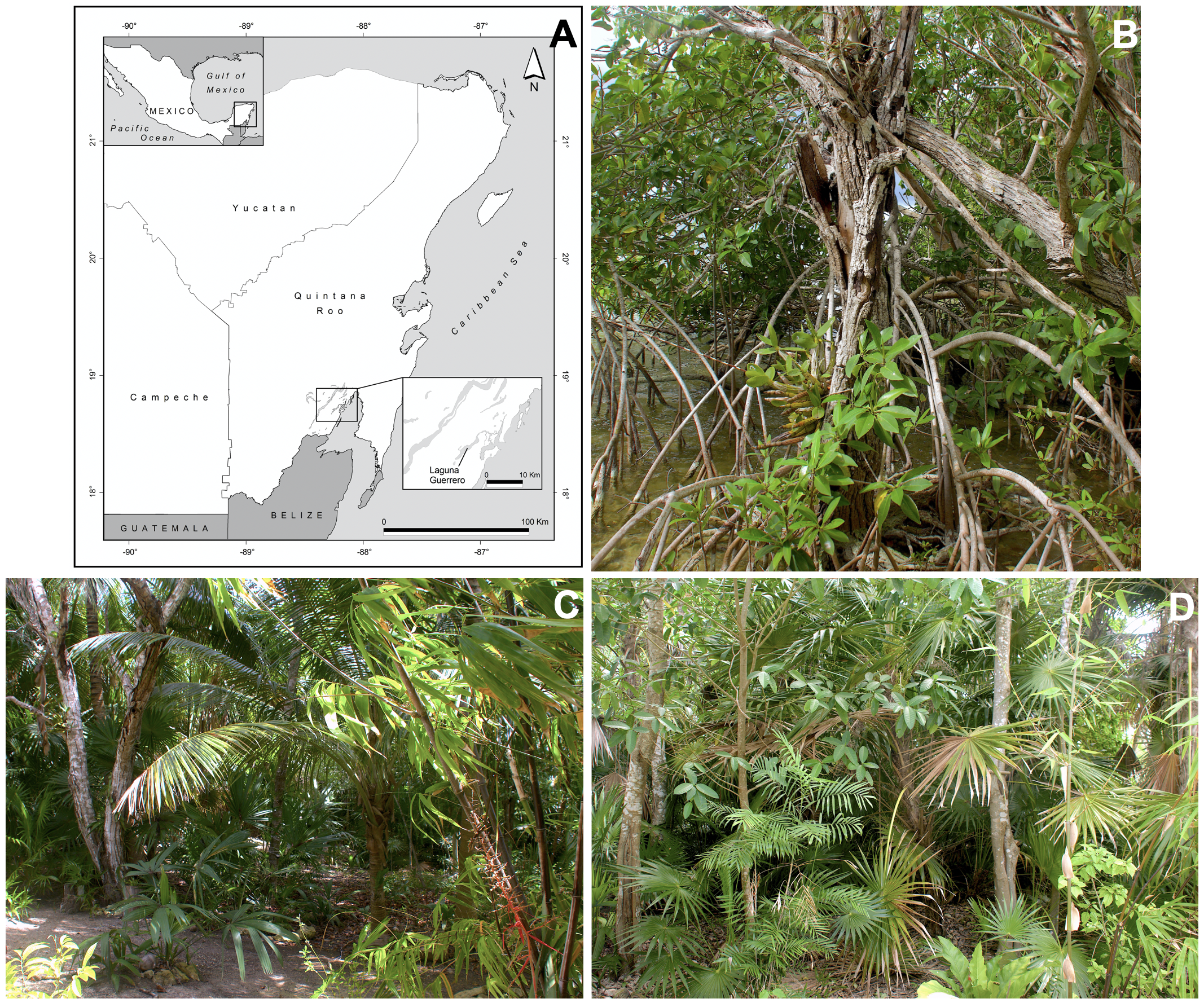

Supplement: Supplemental Information 1 — (A) Location of the study site (Laguna Guerrero) in the southern part of the Yucatan Peninsula, Mexico. (B–D) General view of the habitat. (B) Mangrove (Rhizophora mangle) with epiphytes, namely Myrmecophila tibicinis (bottom, near center). (C) and (D) Examples of indigenous trees (Lysolima latisiliquum, Manilkara zapota, Guazuma ulmifolia), indigenous palm trees (Thrinax radiata), intermixed with coconut palm trees (Cocos nucifera) and ornamental plants (black bamboo, Phyllostachys nigra). Map credit: Holger Weissenberger. Photos credit: Jean-Paul Lachaud. [file peerj-09-11949-s001.png]

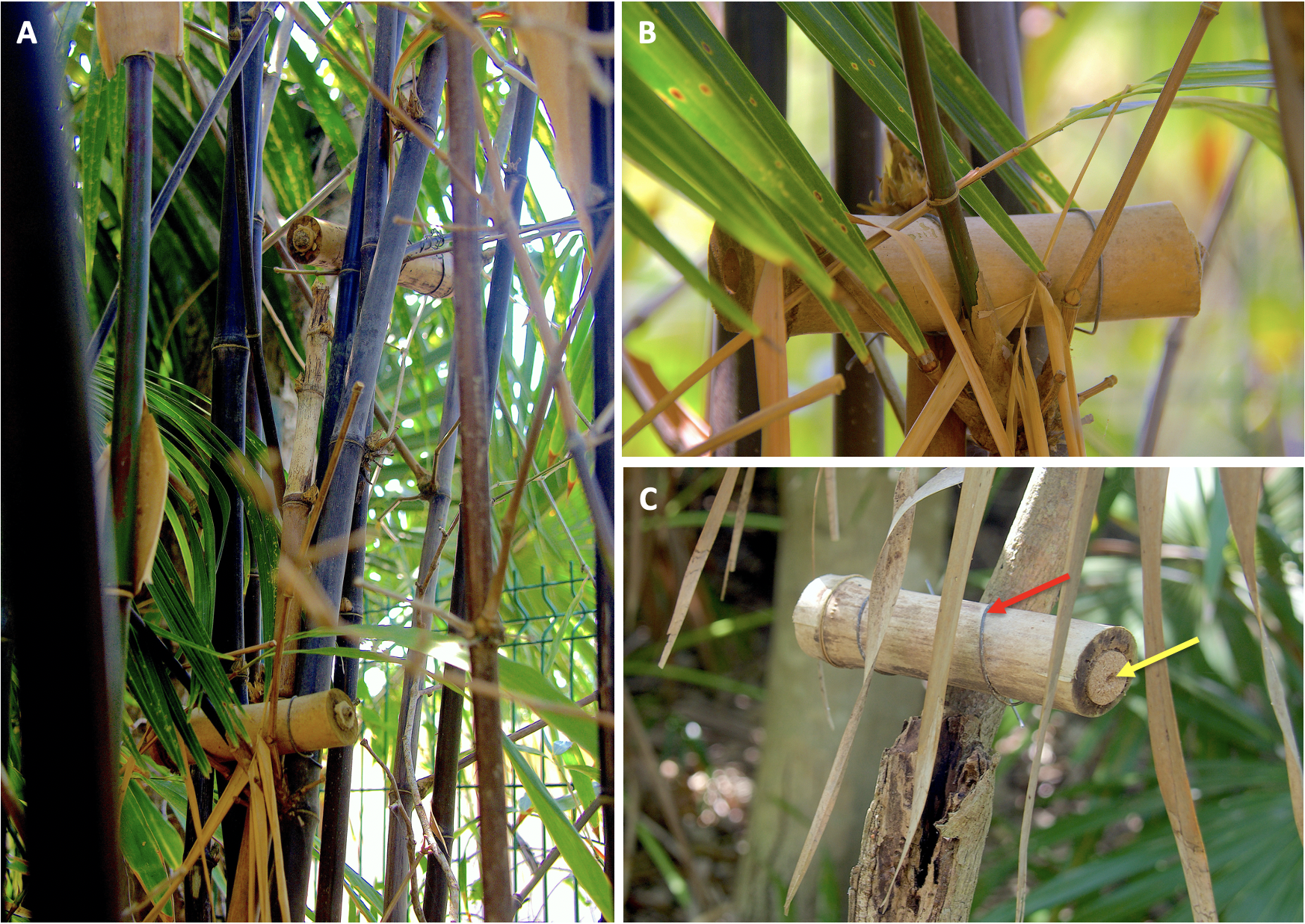

Supplement: Supplemental Information 2 — (A) Position of the two nesting units corresponding to sample #6CC. (B) Close-up of the lowest unit of sample #6CC. (C) Details of the nest entrance and the attachment of an artificial nest at another location. We used hollow bamboo internodes drilled at one end and closed with a cork pierced with a hole, which served as the entrance (yellow arrow in C). These artificial nests were then attached with wires (red arrow in C), at a heigh of at least 1.2 m, to a plant support where foragers had been previously observed and left without intervention for four weeks. Prior to the collection of sample #6CC, numerous interactions were observed between the two artificial nests, with several workers going back and forth between the two nesting units. Photos credit: Jean-Paul Lachaud. [file peerj-09-11949-s002.png]

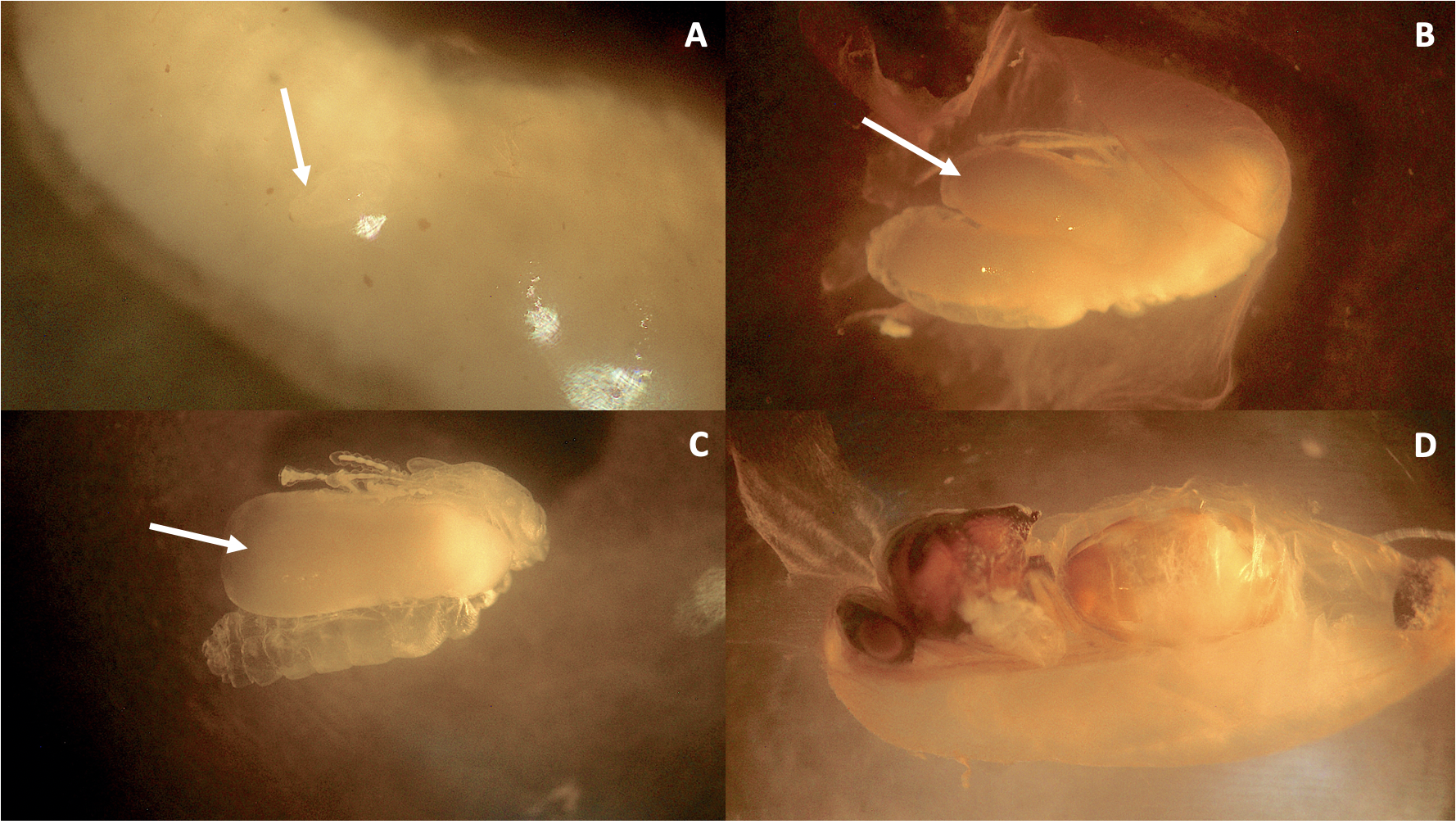

Supplement: Supplemental Information 3 — (A) Unidentified fully fed planidium upon a C. rectangularis prepupa. Arrow points at the swollen planidium, barely visible. (B) Unidentified L3 (arrow) upon C. rectangularis pupa. (C) Unidentified late L3-prepupa (arrow) upon the host remains. (D) Pupa of the female Obeza sp. The host cocoon has been removed in A and C. Photos credit: Gabriela Pérez-Lachaud. [file peerj-09-11949-s003.png]

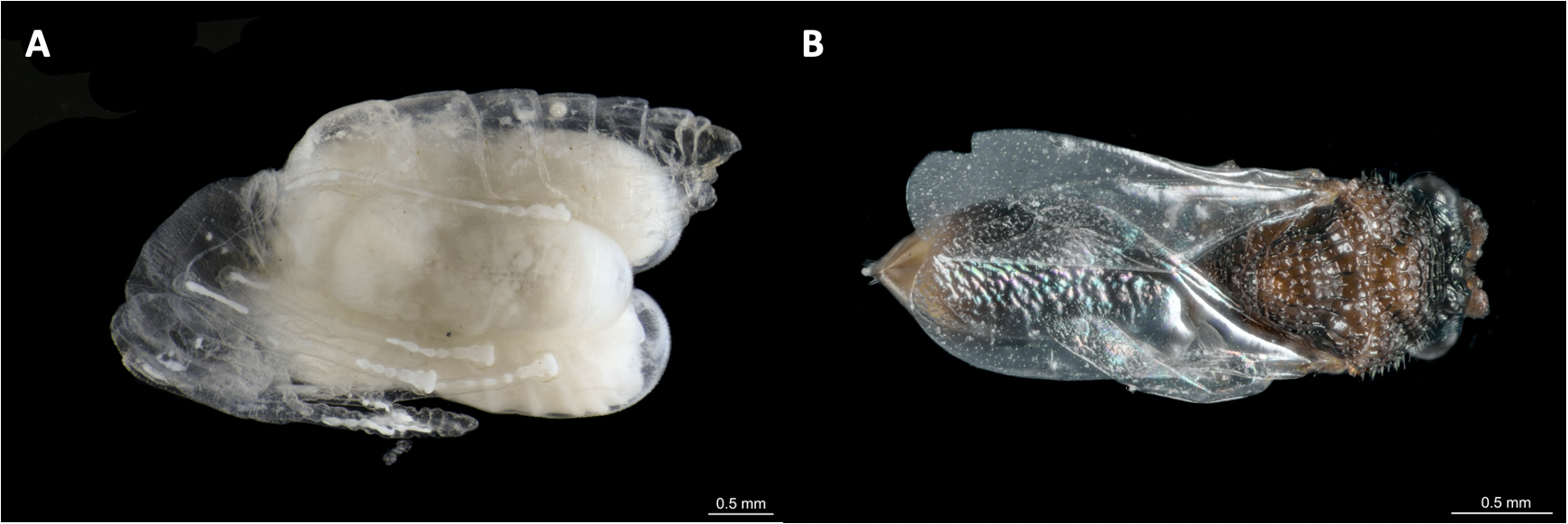

Supplement: Supplemental Information 4 — (A) Three late L3-prepupae developing upon a single host. (B) Female, dorsal view. The host cocoon has been removed. Photos credit: Humberto Bahena-Basave. [file peerj-09-11949-s004.png]
